# A nanotheranostic agent based on Nd^3+^-doped YVO_4_ with blood-brain-barrier permeability for NIR-II fluorescence imaging/magnetic resonance imaging and boosted sonodynamic therapy of orthotopic glioma

**DOI:** 10.1038/s41377-022-00794-9

**Published:** 2022-04-29

**Authors:** Zhijia Lv, Longhai Jin, Yue Cao, Hao Zhang, Dongzhi Xue, Na Yin, Tianqi Zhang, Yinghui Wang, Jianhua Liu, Xiaogang Liu, Hongjie Zhang

**Affiliations:** 1 State Key Laboratory of Rare Earth Resource Utilization, Changchun Institute of Applied Chemistry (CIAC), Chinese Academy of Sciences, 130022 Changchun, China; 2 University of Science and Technology of China, 230026 Hefei, Anhui China; 3 Ganjiang Innovation Academy, Chinese Academy of Sciences, 341000 Ganzhou, Jiangxi China; 4 Department of Radiology, The Second Hospital of Jilin University, 130041 Changchun, China; 5 Department of Neurosurgery, The First Hospital of Jilin University, 130041 Changchun, China; 6 Department of Chemistry, National University of Singapore, Singapore, 117543 Singapore; 7 Department of Chemistry, Tsinghua University, 100084 Beijing, China

**Keywords:** Imaging and sensing, Nanoparticles

## Abstract

The specific diagnosis and treatment of gliomas is a primary challenge in clinic due to their high invasiveness and blood-brain barrier (BBB) obstruction. It is highly desirable to find a multifunctional agent with good BBB penetration for precise theranostics. Herein, we design and construct a core-shell structured nanotheranostic agent (YVO_4_:Nd^3+^-HMME@MnO_2_-LF, marked as YHM) with YVO_4_:Nd^3+^ particles as the core and MnO_2_ nanosheets as the shell. Sonosensitizer hematoporphyrinmonomethyl ether (HMME) and lactoferrin (LF) were further loaded and modified on the surface, giving it a good ability to cross the BBB, near-infrared fluorescence imaging in the second window (NIR-II)/magnetic resonance imaging (MRI) bimodality, and highly efficient sonodynamic therapy (SDT) of orthotopic gliomas. The YVO_4_:Nd^3+^ (25%) core exhibited good NIR-II fluorescence properties, enabling YHM to act as promising probes for NIR-II fluorescence imaging of vessels and orthotopic gliomas. MnO_2_ shell can not only provide O_2_ in the tumor microenvironments (TME) to significantly improve the healing efficacy of SDT, but also release Mn^2+^ ions to achieve T_1_-weight MRI in situ. Non-invasive SDT can effectively restrain tumor growth. This work not only demonstrates that multifunctional YHM is promising for diagnosis and treatment of orthotopic glioma, but also provides insights into exploring the theranostic agents based on rare earth-doped yttrium vanadate nanoparticles.

## Abstract (web summary)

Nd^3+^-doped YVO_4_ nanotheranostic agent with blood-brain-barrier permeability could achieve NIR-II imaging/MRI and efficient SDT of orthotopic gliomas.

## Graphical abstract

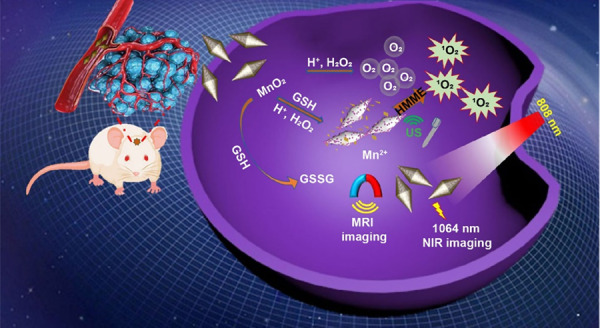

## Introduction

Glioma is an intracranial malignant tumor that still poses a major clinical challenge due to its highly invasive nature, low cure rate, and high mortality rate^1–3^. Although advanced molecular imaging techniques can be used for glioma diagnosis, their low spatial resolution, harmful ionization, and complicated workflow limit their application in real-time intraoperative imaging^4–6^. In recent years, fluorescence imaging technology, especially in the second near-infrared bio-window (NIR-II, 1000–1700 nm) has aroused much more interest because of its unique advantages, including high spatial resolution, low tissue absorption and scattering, and dynamic real-time imaging^7–10^. In the last 10 years, numerous fluorescent nanomaterials have been exploited as NIR-II fluorescence imaging probes^11–15^. Among them, rare earth-doped luminescent nanoparticles (RELNs) are recognized promising probes thanks to their good photostability, large Stokes shift, and long lifetime^16,17^. Since the discovery of the inhibitory effect on ATPase and insulin-like effect, scientists have been encouraged to focus on the biological study of vanadium compounds. Moreover, the vanadate has many merits for great potential biological applications, such as the simple preparation method, and controllable size. Yttrium vanadate (YVO_4_) with low-energy phonons is one of the most intensively investigated host materials for various optical applications, such as phosphors and lasers^18,19^. YVO_4_:Eu^3+^ nanoparticles have been applied as the probes for fluorescent imaging in the visible range^20^. Compared with the fluorescent probe in the visible range, it is highly desired to explore the YVO_4_ nanoparticles with the good NIR-II luminescence property excited by NIR light. Nd^3+^ ions have a series of absorption bands in the NIR region, which can act as not only sensitizer to transfer the energy to Yb^3+^ and Er^3+^ ions, but also activator for NIR-II fluorescent imaging. Therefore, Nd^3+^ doped YVO_4_ nanophosphors have great potential in bioimaging applications. This making Nd^3+^-doped YVO_4_ nanophosphors particularly attractive for bioimaging. However, there is no report on the use of Nd^3+^-doped YVO_4_ nanophosphors for bio-applications, let alone for bioimaging in orthotopic gliomas.

As for the glioma therapy, current treatment focuses on surgical resection, chemotherapy, and radiotherapy, but five-year survival rate of no more than 5% is mainly due to the fact that lesions are rarely completely removed and most drugs and contrast agents cannot enter the tumor through the blood-brain barrier (BBB)^21,22^. Consequently, it is extremely important to develop new treatments for gliomas. Sonodynamic therapy (SDT) that produced reactive oxygen species (ROS) under ultrasound excitation, is a promising therapy for gliomas because it penetrates deep into the tumor, is non-invasive, does not emit radiation, and has few side effects on normal tissue^23–25^. However, SDT consumes oxygen in the tumor microenvironment (TME) and exacerbates hypoxia. Therefore, increasing the oxygen supply to the tumor is a good strategy to improve the curative effect^26–28^. MnO_2_ nanomaterials are of particular interest as TME-responsive O_2_ producers since they can catalyze with H_2_O_2_ in the TME to yield oxygen, which both improves the efficacy of treatment and relieves hypoxia. Furthermore, free Mn^2+^ ions act as excellent contrast agent for T_1_-weighted magnetic resonance imaging (MRI)^29,30^. Consequently, constructing the novel multifunctional nanotheranostic agents by integrating YVO_4_:Nd^3+^ with MnO_2_ is a potential approach for bimodal NIR-II imaging/MRI-guided SDT of orthotopic gliomas.

**Scheme 1:**
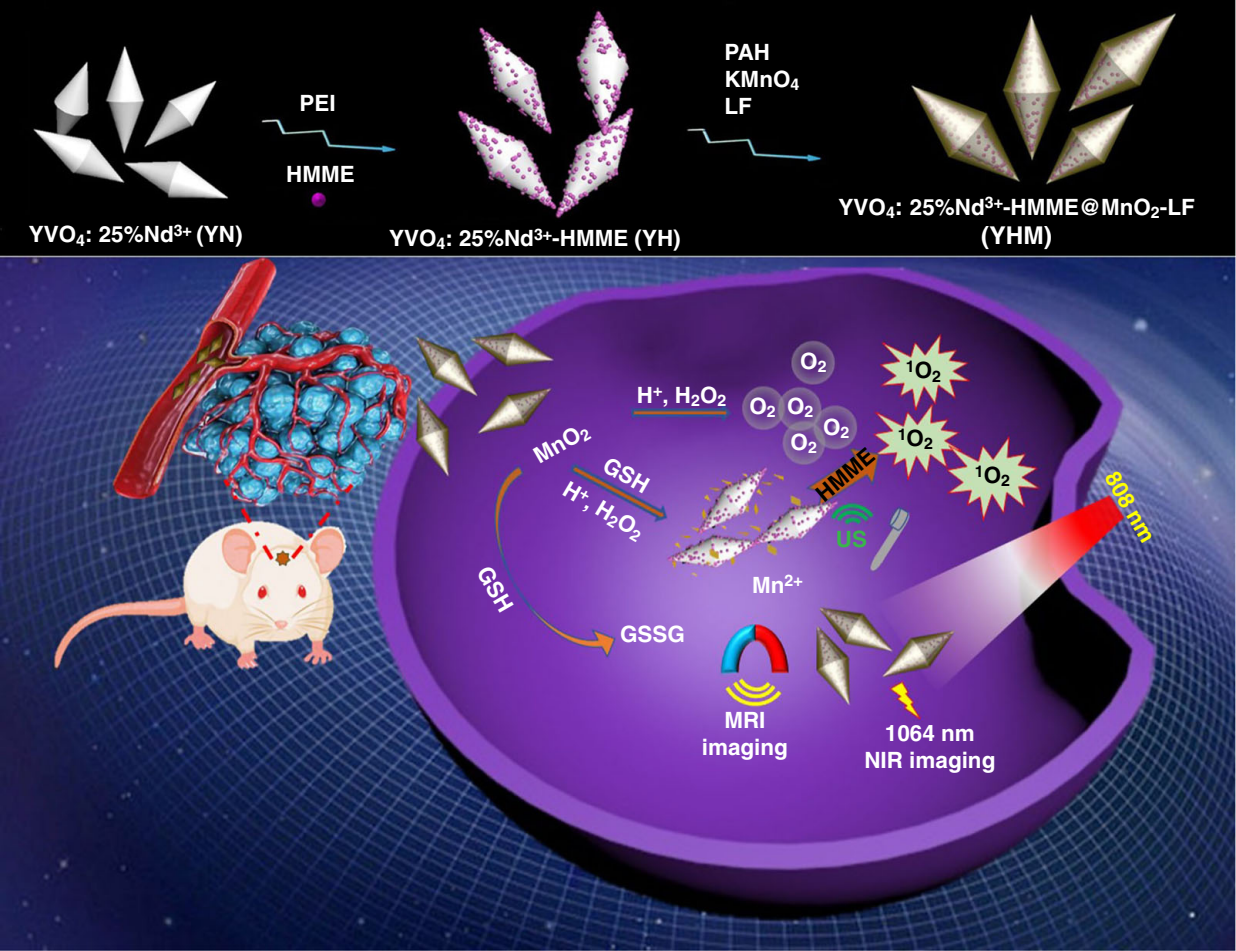
Schematic drawing of TME self-enhanced SDT and multimodal bioimaging

Herein, we prepared a multifunctional nanotheranostic agent with YVO_4_:Nd^3+^ particles as the core and the carrier of sonosensitizer hematoporphyrinmonomethyl ether (HMME) and MnO_2_ nanosheets as the shell for NIR-II imaging/MRI and high-efficiency SDT of orthotopic gliomas (Scheme 1). Then lactoferrin (LF) was further modified on its surface due to the over-expressed lactoferrin receptor in glioma cells, endowing YVO_4_:Nd^3+^-HMME@MnO_2_-LF (designated as YHM) with good targeting and transmittance of the BBB^22,31–33^. The YHM exhibits strong emission peak at 1064 nm under 808 nm laser, enabling NIR-II fluorescence imaging. In TME, the MnO_2_ shell can not only catalyze the disintegration of H_2_O_2_ to release O_2_, which further enhances the generation of ^1^O_2_ under US radiation, but also releases Mn^2+^ for TME-responsive MRI. All these findings make YHM as a great potential theranostic nanoagent for NIR-II imaging/MRI and TME self-enhanced SDT of orthotopic gliomas.

## Results

**Fig. 1:**
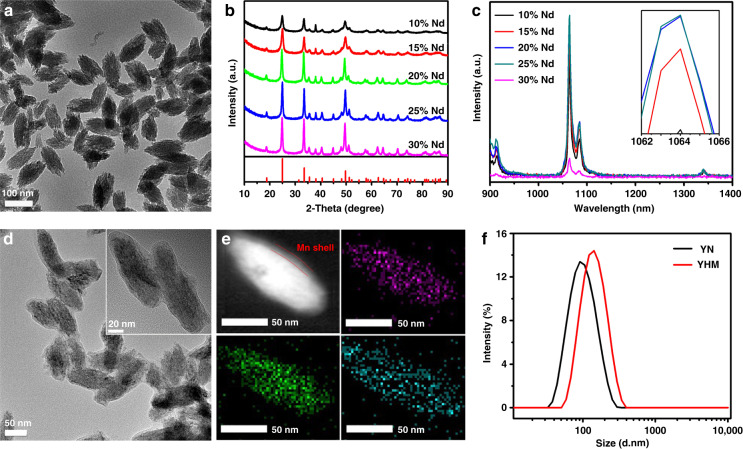
The structure characterization of YN and YHM. **a** TEM image of YN. **b** XRD spectra of different concentrations of Nd^3+^-doped YVO_4_. **c** Fluorescence spectra of YVO_4_ with different rare earth contents under 808 nm excitation (0.4 W cm^-2^) (the inset is enlarged 1064 nm emission peak). **d** TEM image of YHM (the figure is a high-magnification image). **e** Corresponding HAADF-STEM image of YHM and elemental distribution mapping of Y (purple), V (green) and Mn (indigo). **f** Dynamic light scattering (DLS) of YN and YHM

YVO_4_:Nd^3+^ particles were first synthesized by the coprecipitation method and exhibited a uniform and monodisperse spindle-shaped morphology (Fig. 1a). X-ray diffraction (XRD) patterns showed that all YVO_4_:Nd^3+^ particles with different doping concentrations of Nd^3+^ ions had a tetragonal structure (JCPDS card No. 01-070-1281, Fig. 1b). Typical emissions of Nd^3+^ at 1064 nm and 1340 nm were observed, corresponding to the ^4^F_3/2_ → ^4^I_11/2_ and ^4^F_3/2_ → ^4^I_13/2_ transitions of Nd^3+^, respectively^34^. When the doped Nd^3+^ concentration reached 25%, the strongest emissions were obtained, so the optimal YVO_4_: 25% Nd^3+^ (YN) was used for the following experiments (Fig. 1c). Then the sonosensitizer HMMEs were loaded onto the surface of YVO_4_:25% Nd^3+^ (YVO_4_: 25% Nd^3+^-HMME, labeled as YH), and further coated MnO_2_ shell and functionalized LF molecules. The YHM exhibits an obvious core-shell structure as shown in Fig. 1d, demonstrating by the high angle annular dark-field scanning transmission electron microscopy (HAADF-STEM) image and elemental mapping. The Y and V are mainly distributed in the core, and Mn shell is visible (Fig. 1e). The appearance of characteristic peaks of Mn 2p_1/2_ and Mn 2p_3/2_ in the spectrum of X-ray photoelectron spectroscopy (XPS), confirming successful cladding MnO_2_ shell (Fig. S1)^35^. The UV–Vis absorption spectra of YH and YHM show a clear absorption peak of HMME at 397 nm, demonstrating the efficient loading of HMME (Fig. S2)^23^. In Fig. 1f, after functionalization with LF by electrostatic interaction, YHM exhibits good dispersibility in water with the hydrodynamic diameter of about 140 nm. The changed zeta potential also proves that the desired structure is achieved at each step (Fig. S3).

**Fig. 2:**
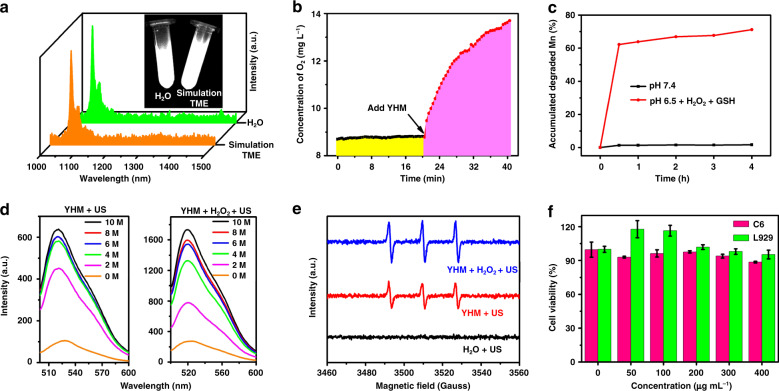
The properties evaluation of YHM. **a** Fluorescence spectra of YHM in water and simulated TME (the inset shows the corresponding NIR-II images). **b** Oxygen production capacity curve of H_2_O_2_ and YHM + H_2_O_2_. **c** Mn release curves of YHM under simulated TME and pH=7.4 PBS. **d** ^1^O_2_ generation capacity curve (SOSG probe) of YHM + US and YHM + H_2_O_2_ + US groups. **e** Detection of ^1^O_2_ generation under different conditions by ESR spectra. **f** Cytotoxicity of L929 cells and C6 cells under different concentrations of YHM

Because the MnO_2_ shell could release Mn^2+^ and produce O_2_ in TME by the following reactions^30^:

$${{{\mathrm{MnO}}}}_2 + 2{{{\mathrm{H}}}}^ + \to {{{\mathrm{Mn}}}}^{2 + } + {{{\mathrm{H}}}}_2{{{\mathrm{O}}}} + 1/2{{{\mathrm{O}}}}_2 \uparrow$$

$${{{\mathrm{MnO}}}}_2 + 2{{{\mathrm{H}}}}^ + + {{{\mathrm{H}}}}_2{{{\mathrm{O}}}}_2 \to {{{\mathrm{Mn}}}}^{2 + } + 2{{{\mathrm{H}}}}_2{{{\mathrm{O}}}} + {{{\mathrm{O}}}}_2 \uparrow$$

$${{{\mathrm{MnO}}}}_2 + 2{{{\mathrm{GSH}}}} + 2{{{\mathrm{H}}}}^ + \to {{{\mathrm{Mn}}}}^{2 + } + 2{{{\mathrm{H}}}}_2{{{\mathrm{O}}}} + {{{\mathrm{GSSG}}}}$$

the photostability of YHM was investigated in TME (100 μM H_2_O_2_ + pH 6.5 + 1 mM GSH). As shown in Fig. 2a, the luminescence intensity of YHM in simulated TME does not change significantly compared to that in aqueous solution, making them suitable as promising contrasts for NIR-II fluorescence imaging. Moreover, the generation of O_2_ is detected with a portable dissolved oxygen meter (Fig. 2b). After the addition of 200 ppm YHM into H_2_O_2_, a large amount of O_2_ was produced, indicating its good ability to alleviate the hypoxia of TME. In the normal condition (pH = 7.4, phosphate-buffered saline (PBS)), few Mn^2+^ ions were released from YHM, indicating the good stability during circulation. The core-shell structure of YHM was retained after mixing with PBS and fetal bovine serum (FBS) for 48 h (Fig. S4). In contrast, nearly 71.14% Mn^2+^ ions were released from YHM within 4 h in the simulated TME, giving YHM the potential as a TME-responsive MRI contrast agent. Next, the ^1^O_2_ generation capacity of the sonosensitizer HMME was tested upon irradiation with US using the Singlet Oxygen Sensor Green reagent (SOSG). The green emission boosted with the extension of US irradiation, indicating the ^1^O_2_ generation (Fig. 2d). After the addition of H_2_O_2_, the ability to generate ^1^O_2_ is higher than that of YHM alone, which could be ascribed to the generation of O_2_ to improve the treatment effect of SDT. Moreover, the ^1^O_2_ generation of YHM was verified by the result of electron spin resonance (ESR) spectroscopy (Fig. 2e). Compared to the H_2_O + US group, the ^1^O_2_ signals of YHM + US group were detected, implying the generation of ^1^O_2_. After adding H_2_O_2_, the ^1^O_2_ signal is stronger than that of YHM + US group, further confirming that O_2_ production enhances the yield of ^1^O_2_ and thus promotes SDT. These findings demonstrate that YHM is capable for NIR-II imaging and SDT. In terms of biological applications, the cytotoxicity of YHM was investigated for normal cells (mouse fibroblasts (L929)) and C6 rat glioma cells (C6) by CCK-8 assay (Fig. 2f). The YHM nanocomposite showed low cell cytotoxicity to C6 and L929 cells even at high concentration, demonstrating good biocompatibility.

**Fig. 3:**
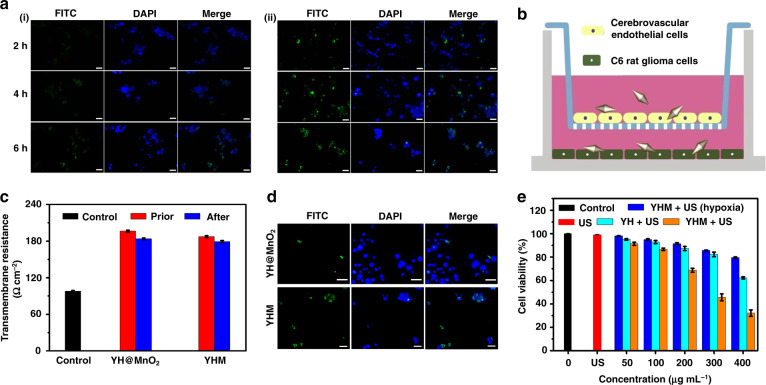
The targeting ability and therapeutic effect of YHM. **a** Staining images of C6 cells incubated with YH@MnO_2_ (i) and YHM (ii) for multiple times. **b** Graphic of BBB model. **c** Trans-endothelial electrical resistance value before and after YHM was traversed by the BBB model. **d** Fluorescent labeling staining of C6 cells after traversing the BBB model. **e** Cell viability of C6 cells under dissimilar conditions, scale bar: 100 μm

The endocytosis efficiency of nanoparticle by tumor cells is a critical factor in the tumor treatment. Therefore, we loaded the fluorescein isothiocyanate isomer (FITC) onto the YH@MnO_2_ (YHM without modification LF) and YHM surface to evaluate their endocytosis efficiency by C6 cells. After co-incubation with YH@MnO_2_ and YHM for 2, 4 and 6 h, the nuclei of C6 cells were marked with 4′,6-diamidino-2-phenylindole (DAPI). In Fig. 3a, a small amount of YH@MnO_2_ was endocytosed even after incubation for 6 h. In contrast, the green fluorescence of FITC was seen in the cytoplasm of the YHM group, and the signal intensity enlarged with increasing duration, indicating the modification of LF endows YHM with good targeting ability for C6 cells, and allows them to enter cells more effectively. It is crucial that the nanodrug can pass through the BBB, which is the basis for further application in vivo. Thus, the BBB model was constructed to verify the ability of YHM to penetrate the BBB. Cerebrovascular endothelial cells (b. End3 cells) proliferated in the upper trans-well chamber to simulate the BBB, and C6 cells were grown in lower trans-well chamber to endocytose YHM NPs (Fig. 3c)^36^. Successful establishment of the BBB was proved by the values of trans-endothelial electrical resistance (TEER), which exceeded 180 Ω cm^-2^ (Fig. 3d)^37^. Then, FITC was loaded to the surface of YH@MnO_2_ and YHM to evaluate their ability to penetrate the BBB. The C6 cells in the lower chamber showed green fluorescence when YHM was added to the upper chamber for 24 h (Fig. 3e), indicating that YHM penetrated the BBB. In contrast, the YH@MnO_2_ group exhibited weak green fluorescence under the same conditions. The result confirms that LF can assist YHM to cross the BBB. The quantitative estimation of the BBB penetration capacity of YHM and YH@MnO_2_ is further studied by inductively coupled plasma mass spectrometry (ICP-MS). Nearly 17.5% YHM could be detected in the bottom, which is 4.5 times higher than that of YH@MnO_2_ in the same condition (Fig. S5). There is no large fluctuation of TEER after YHM passes the BBB, demonstrating that the simulated BBB still remains tight (Fig. 3d). All these results confirm that YHM, with its good ability to penetrate the BBB and target C6 cells, has great potential for further applications.

**Fig. 4:**
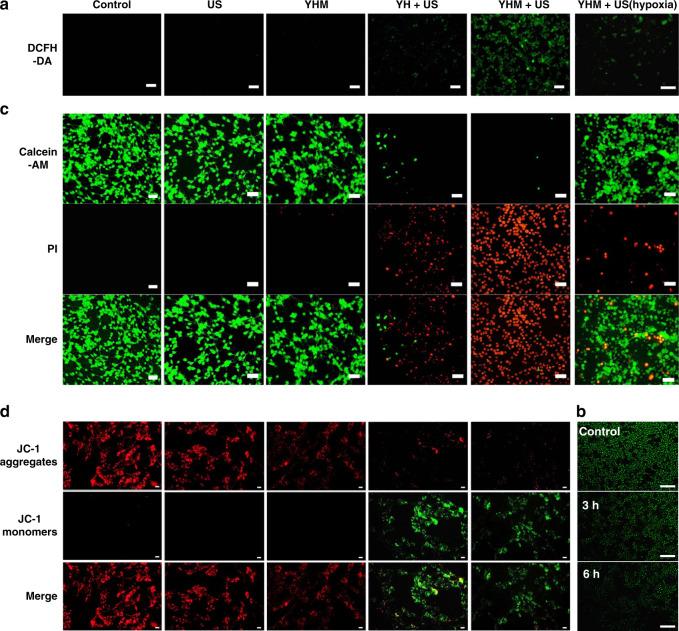
The properties evaluation of YHM in vitro. Staining images of DCFH-DA (**a**), calcein-AM&PI assay (**c**) and mitochondrial membrane potential (**d**) for C6 cells in different groups; (**b**) [Ru(dpp)_3_]Cl_2_ probe labeled YHM + H_2_O_2_ for intracellular oxygen staining at different timepoints, scale bar: 100 μm

We further investigated the treatment effect of YH and YHM under US irradiation. As shown in Fig. 3e, in contrast to the US group, the survival rate of the YH and YHM group decreased with increasing concentration after 4 min of US irradiation. Furthermore, the killing effect of YHM was significantly higher than that of YH under the same conditions, which may be because the O_2_ production of YHM in TME promotes the production of ^1^O_2_. The YHM + US (hypoxia) group also showed the inhibition of cell viability, further verifying the ^1^O_2_ production owing to the generation of O_2_ from YHM in the TME. This result is further confirmed by the generation of ^1^O_2_ in C6 cells using 2’,7’-dichlorodihydrofluorescein diacetate (DCFH-DA). In contrast to control, US and YHM groups, the weak green emission was detected in the YH + US group, indicating production of ^1^O_2_ due to the loading of HMME (Fig. 4a).

The YHM + US group showed the stronger green emission, demonstrating that the generation of O_2_ by the MnO_2_ shell in TME can enhance the ^1^O_2_ production. Even if in the hypoxia condition, the faint green fluorescence signal also can be detected, verifying the ability of YHM for relieving tumor hypoxia. The intracellular oxygen production was further verified by using [Ru(dpp)_3_]Cl_2_ as probe. The intensity of green fluorescence decreased with time extension after YHM co-incubated with H_2_O_2_, which proved the generation of O_2_ in C6 cells (Fig. 4b). From the calcein AM and propidium iodide (PI) co-staining results, nearly no death cells were observed in the control, US and YHM group. In contrast, inhibiting effect of YHM + US group is better than that of YH + US group and YHM + US (hypoxia) group, which is in accordance with the above results (Fig. 4c). Apoptotic process was often accompanied with mitochondria dysfunction, thus the mitochondria membrane potential was investigated by JC-1 staining (Fig. 4d). In contrast to the control, US and YHM groups, the obvious green fluorescence can be observed in YH + US and YHM + US group, demonstrating the low mitochondrial membrane potential and mitochondria dysfunction. The YHM + US group displayed stronger green fluorescence than YH + US group, indicating that the participation of oxygen indeed promoted the SDT and resulted in more cell apoptosis.

**Fig. 5:**
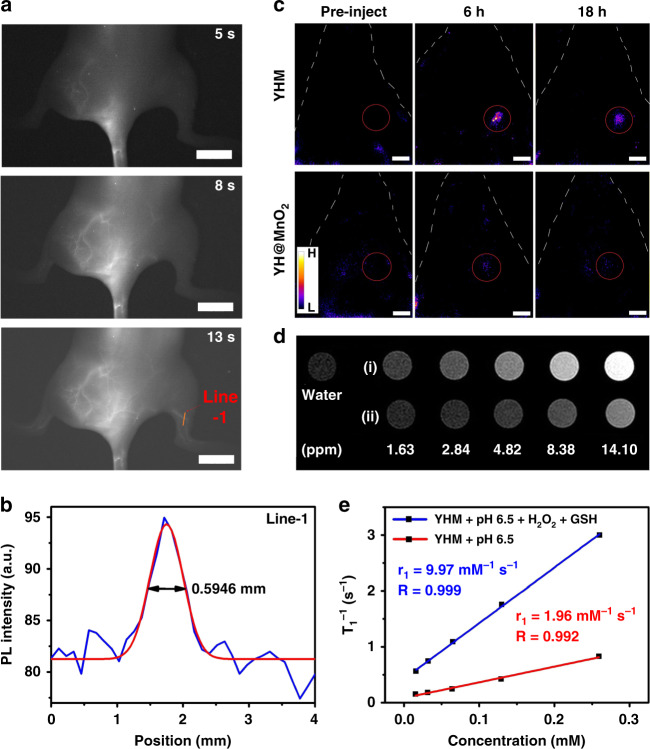
The NIR-II imaging and MRI of YHM. **a** Vascular NIR-II images showing hindlimb of a C57BL/6J mouse (scale bar: 10 mm). **b** Measurement of the vessel width of the lateral thigh shown in (**a**) (orange solid line). **c** NIR-II imaging of C57BL/6 J mice orthotopic gliomas after injection of YHM and YH@MnO_2_ via the tail vein, scale bar: 5 mm. **d** T_1_-weighted in vitro MRI images of YHM compared with PBS ((i) pH 6.5 + 100 μM H_2_O_2_ + 1 mM GSH, (ii) pH 6.5). **e** Relaxation rate r_1_ versus the concentration of Mn

It is crucial to obtain comprehensive and accurate information about gliomas for diagnosis and tailored treatment. The feasibility of YHM as NIR-II fluorescence imaging/MRI bimodal imaging contrast agent was further investigated. We first evaluated the ability of YHM for vascular NIR-II fluorescence imaging excited by 808 nm laser. NIR-II emission can be observed from the vascular for 5 s after injection with YHM via the tail vein, which benefits from its good luminescence performance in NIR-II window (Fig. 5a). Following blood flow, the vascular network and branches were increasingly clearly imaged, and the Gaussian-fitted full width at half-maximum (FWHM) of the cross-sectional intensity profile was 0.5946 mm, showing the excellent spatial resolution (Fig. 5b)^16^. Then, the orthotopic brain tumor models were constructed by inoculating GL261 cells into C57BL/6J mice to evaluate the capacity of YHM for gliomas diagnosis in vivo. At 6 h post-injection of YHM and YH@MnO_2_ (YHM without LF), the tumor in both two groups was illuminated by NIR-II emission. And the emission intensity of YHM is much higher than that of YH@MnO_2_, further demonstrating that modification with LF molecules gives YHM with good targeting ability for gliomas (Fig. 5c). Therefore, YHM is a promising NIR-II probe for vascular mapping with good spatial resolution and orthotopic gliomas.

Due to the release of paramagnetic Mn^2+^ from YHM in TME, we further assessed the ability of YHM as TME-responsive T_1_-weighted MRI contrast agent. In contrast to normal conditions, the signals in simulated TME increased with increasing Mn concentration (Fig. 5d). The longitudinal relativity r_1_ of YHM in simulated TME was measured to 9.97 mM^−1^ s^−1^, which increased by 5 times compared with normal conditions (Fig. 5e). Then the T_1_-weighted MRI of orthotopic glioma in vivo was further investigated by intravenous injection of YHM (Fig. S6). After injection for 24 h, the MRI signals at tumor position became stronger, making T_1_-weighted MRI of orthotopic gliomas responsive to TME.

**Fig. 6:**
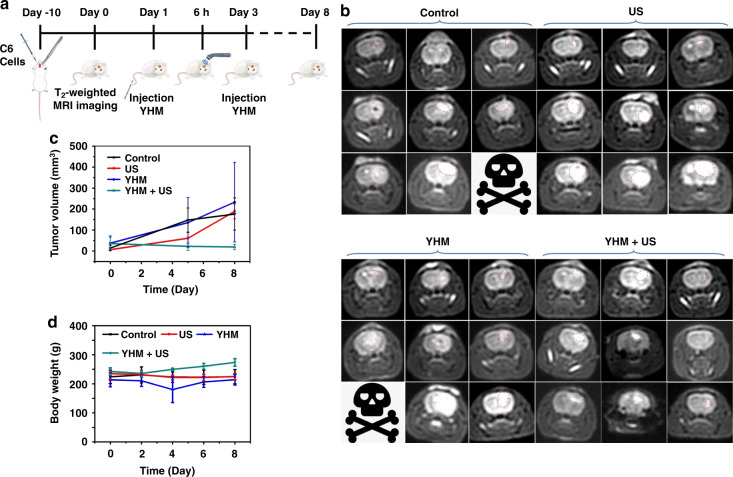
The treatment of orthotopic gliomas. **a** Schematic of cycle and treatment monitoring. **b** The T_2_-weighted MRI images of Control, US, YHM and YHM + US groups at 0, 5 and 8 days. **c** Glioma volume and (**d**) weight of rats in all groups (*n* = 3)

Inspired by the good performance in vitro, orthotopic glioma models were constructed by the inoculation of C6 cells into caudate nucleus of rats to evaluate the treatment effect of YHM in vivo. The biodistribution of YHM in orthotopic gliomas and main organs was studied using ICP-MS (Fig. S7). After injection for 6 h, nearly 10% YHM enriched in the tumor, which determined the time point of US treatment (Fig. 6a). The rats were separated randomly into four groups: Control group, US group, YHM group, and YHM + US group. The size of tumor was assessed by T_2_-weighted MRI. In Fig. 6b and c, the tumors of Control, US and YHM groups grew rapidly during treatment, resulting in the death of individual rats. In contrast, the tumor growth was successfully inhibited in the YHM + US group, which further evidenced the good treatment effect of SDT. Moreover, the weight of rats showed no obvious alteration during treatment (Fig. 6d). The hematoxylin and eosin (H&E) staining of main organs, blood biochemistry, and hemolysis analysis results were further performed to estimate the biocompatibility of YHM. The tissues of main organs did not show distinct differences between the normal rat and tumor-bearing rat after SDT, indicating the good biocompatibility of YHM (Fig. S8). Compared to the blood indexes of normal rat, there are no significant changes in those of the tumor-bearing rats after SDT and the rats after injection YHM for 30 days, which further proved the good long-term safety of YHM in vivo (Fig. S9). The hemolysis rate of YHM is in the safe range, verifying the low effect on hemolysis (Fig. S10). The good treatment effect and high biocompatibility endow YHM with good potential for effective SDT of orthotopic gliomas with low side effects.

## Discussion

We have constructed the nanotheranostic agents YHM for NIR-II imaging/MRI bimodal imaging and highly efficient SDT of orthotopic glioma. YHM exhibited good ability of BBB penetration and specific targeting gliomas in vitro and in vivo due to the functionalization of LF. The YVO_4_: 25% Nd^3+^ core with good NIR-II fluorescence performances enabled YHM to act as promising NIR-II fluorescent probes for blood vessels mapping and orthotopic glioma imaging. MnO_2_ shell could not only generate O_2_, but also release Mn^2+^ ions in TME, which enhanced the treatment effect of SDT and enabled T_1_-weighted MRI imaging. The growth of orthotopic glioma was effectively inhibited by enhanced SDT in vivo. This work reports the exploration of nanotheranostic agents based on rare earth ion-doped yttrium vanadate luminescent nanoparticles for multi-modality imaging and therapy of orthotopic gliomas for the first time, paving the way for the expansion of the application of rare earth ion-doped yttrium vanadate luminescent nanoparticles.

## Materials and methods

### Synthesis of spindle-shaped YVO_4_: Nd^3+^

0.0117 g ammonium vanadate was dissolved in HNO_3_ (1 mL, 4 M), and then Y(NO_3_)_3_ (0.70-0.95 mL, 100 mM) and Nd(NO_3_)_3_ (0.05-0.30 mL, 100 mM) aqueous solutions were dropped into above solution and mixed. The ammonia hydroxide was dropped into the mixed solution until it turned to golden yellow suspension. The solution was continued to sonicate for 1 h until it turned to milky white. Then, centrifuged and washed with ultrapure water. Finally, the spindle-shaped YVO_4_: Nd^3+^ was obtained.

### Synthesis of YVO_4_: 25% Nd^3+^-HMME (YH)

The aqueous solution mixed by YVO_4_: 25% Nd^3+^ (1 mg mL^−1^, 10 mL) and PEI (10 mg mL^−1^, 5 mL) was stirred for 4 h, and centrifuged and washed 3 times with ultrapure water. The obtained nanoparticles were re-dispersed into 10 mL ultrapure water. HMME anhydrous ethanol solution (0.25 mg mL^−1^, 2 mL) was dropped into the above solution, and continued to stir in the dark for 12 h. Then, centrifuged and collected the supernatant. The UV-Vis spectra of HMME (standard curve) and the supernatant of YH were shown in Fig. S11. According to these plots, about 0.4963 g HMME was loaded on the surface of YN-PEI.

### Synthesis of YVO_4_: 25% Nd^3+^-HMME@MnO_2_-LF (YHM)

PAH (10 mg mL^−1^, 5 mL) and YH were stirred for 4 h, and centrifuged to collect. 2 mg KMnO_4_ was added in YH-PAH aqueous solution. After stirring for 1 h, YVO_4_: 25% Nd^3+^-HMME@MnO_2_ (YH@MnO_2_) was obtained by centrifuge, and then added 4 mg LF. After stirring for 4 h in an ice-water bath, YVO_4_: 25% Nd^3+^-HMME@MnO_2_-LF (YHM) was obtained using a refrigerated centrifuge (12000 rpm, 7 min, 4 °C), and washed with water twice. Finally, YHM dispersed in PBS and stored at 4 °C.

### Detection of singlet oxygen (^1^O_2_)

SOSG methanol solution (33 μL, 100 μM) and YHM solution (2 mL, 100 ppm) were handled with US (0.7 W cm^−2^, 3 MHz, 50% duty cycle) for different times, and then luminescent spectra were detected under the 448 nm excitation.

### Cellular endocytosis

200 ppm YH@MnO_2_ and YHM (labeled with FITC) were co-incubated with C6 cells for 2, 4, and 6 h, and washed with PBS twice. 5 ppm DAPI was used to stain cell nuclei and washed with PBS to remove redundant dye. Fluorescent microscope was used to obtain the images.

### In vitro BBB model

1 × 10^6^ b. End3 cells were incubated in 12-well trans-well plate upper chamber (polycarbonate membrane, 0.4 μm pore size) for 4–7 days, until the transmembrane resistance value is higher than 180 Ω cm^−2^ measured by MERS00002 | Millicell ERS-2 Voltohmmeter. After 4.2 × 10^5^ C6 cells were cultured in trans-well lower chamber for 24 h, 200 ppm YH@MnO_2_ and YHM (labeled with FITC) were added in trans-well upper chamber and co-incubated for 24 h. 5 ppm DAPI was used to stain C6 cells nuclei and washed with PBS to remove redundant dye. Fluorescent microscope was used to obtain the images.

### Detection of intracellular singlet oxygen and oxygen production

After incubated with YHM (200 ppm) for 24 h in a 24-well plate, C6 cells were stained with DCFH-DA. After treated with US (0.7 W cm^−2^, 3 MHz, 50% duty cycle, 4 min,1.0 cm thickness pork), the staining images were collected by fluorescent microscope.

C6 cells were incubated for 24 h in a 96-well plate, and [Ru(dpp)_3_]Cl_2_ (Luminescent oxygen sensor, 1 μM) was added. After washed with PBS, 100 μM H_2_O_2_ and 200 ppm YHM were added and co-cultured for different times. Washed with PBS and collected images by fluorescent microscope.

### Animal orthotopic glioma model

All of the animal experiments were conducted according to the rules of the Institutional Animal Care and Use Committee of Tsinghua University (IACUC, 20200330005).

The orthotopic glioma models were constructed with the inoculation of C6 cells (4.2 × 10^6^ in 7 μL PBS) into the caudate nucleus of Rattus norvegicus (6–8 weeks, femina). The location was bregma +0.5 mm, right lateral 3.0 mm, depth 5.0 mm and return to 3.0 mm. GL261 mouse glioma cells (GL261 cells, 5.6 × 10^6^ in 7 μL PBS) were pushed into the caudate nucleus of C57BL/6J mice for establish gliomas model. The location was bregma +1.0 mm, right lateral 2.0 mm, profundity 3.0 mm and return to 2.5 mm.

## Supplementary information

Supporting Information for A nanotheranostic agent based on Nd3+-doped YVO4 with blood-brain-barrier permeability for NIR-II fluorescence imaging/magnetic resonance imaging and boosted sonodynamic the
