# Supplementary material for: A nanotheranostic agent based on Nd3+-doped YVO4 with blood-brain-barrier permeability for NIR-II fluorescence imaging/magnetic resonance imaging and boosted sonodynamic therapy of orthotopic glioma
Source: Light Sci Appl. 2022 Apr 29;11:116. doi: 10.1038/s41377-022-00794-9 (PMC9055055; doi:10.1038/s41377-022-00794-9)
Supplement: Supplementary file 1 — Supporting Information for A nanotheranostic agent based on Nd3+-doped YVO4 with blood-brain-barrier permeability for NIR-II fluorescence imaging/magnetic resonance imaging and boosted sonodynamic the [file 41377_2022_794_MOESM1_ESM.docx]

**Supporting Information for**

**A nanotheranostic agent based on Nd^3+^-doped YVO_4_ with blood-brain-barrier permeability for NIR-II fluorescence imaging/magnetic resonance imaging and boosted sonodynamic therapy of orthotopic glioma**

Zhijia Lv^a, b, c^, Longhai Jin^d^, Yue Cao^e^, Hao Zhang^a, b^, Dongzhi Xue^a, b^, Na Yin^a, b^, Tianqi Zhang^d^, Yinghui Wang^a, b*^, Jianhua Liu^d,*^, Xiaogang Liu^f^, Hongjie Zhang^a, b, c, g*^

^a^ State Key Laboratory of Rare Earth Resource Utilization, Changchun Institute of Applied Chemistry (CIAC), Chinese Academy of Sciences, 130022, Changchun, China

^b^ University of Science and Technology of China, 230026, Hefei, Anhui, China

^c^ Ganjiang Innovation Academy, Chinese Academy of Sciences, 341000, Ganzhou, Jiangxi, China

E-mail: [lvzhijia@ciac.ac.cn](mailto:lvzhijia@ciac.ac.cn) (Z.J. Lv); [zhhust@ciac.ac.cn](mailto:zhhust@ciac.ac.cn) (H. Zhang); [xuedongzhi@ciac.ac.cn](mailto:xuedongzhi@ciac.ac.cn) (D.Z. Xue); [yinna@ciac.ac.cn](mailto:yinna@ciac.ac.cn) (N. Yin); [yhwang@ciac.ac.cn](mailto:yhwang@ciac.ac.cn) (Y.H. Wang); [hongjie@ciac.ac.cn](mailto:hongjie@ciac.ac.cn) (H.J. Zhang)

^d^ Department of Radiology, The Second Hospital of Jilin University, 130041, Changchun, China

E-mail: [jinlonghai@jlu.edu.cn](mailto:jinlonghai@jlu.edu.cn) (L.H. Jin); [happytianqi@yeah.net](mailto:happytianqi@yeah.net) (T.Q. Zhang); [drliujh@yahoo.com](mailto:drliujh@yahoo.com) (J.H. Liu)

^e^ Department of Neurosurgery, The First Hospital of Jilin University, 130041, Changchun, China

E-mail: [caoy20@mails.jlu.edu.cn](mailto:caoy20@mails.jlu.edu.cn) (Y. Cao)

^f^ Department of Chemistry, National University of Singapore, 117543, Singapore

E-mail: [xiaogang_liu@sutd.edu.sg](mailto:xiaogang_liu@sutd.edu.sg) (X.G. Liu)

^g^ Department of Chemistry, Tsinghua University, 100084, Beijing, China

**Experimental methods and materials**

**Materials**

Ammonium vanadate (NH_4_VO_3_), Poly (allylamine hydrochloride) (PAH) and Ethylene imine polymer (PEI) were purchased from Aladdin. Nitric acid (HNO_3_) was purchased from Xilong Scientific. Yttrium(Ⅲ) nitrate hexahydrate and lactoferrin (LF) were purchased from Macklin. Neodymium nitride hexahydrate was purchased from Sinopharm chemical reagent. Ammonium hydroxide (NH_3_·H_2_O) was purchased from Tian in fuyu fine chemical. Potassium permanganate (KMnO_4_) was purchased from Beijing chemical works. Hematoporphyrinmonomethyl ether (HMME) was procured from Shanghai yuanye bio-technology Co., Ltd. Singlet oxygen sensor green (SOSG) was attained from Thermo Fisher Scientific Co., Ltd. The Cell Counting Kit-8 (CCK-8) was procured from Changchun Sanbang Pharmaceutical Technology Co., Ltd. Calcein acetoxymethyl ester (Calcein AM), 2,7-dichlorofluorescin diacetate (DCFH-DA) were gained from Sigma-Aldrich. 4',6-diamidino-2-phenylindole (DAPI) and Fluorescein isothiocyanate isomer (FITC) were attained from Beyotime Biotechnology.

**Cytotoxicity of YHM**

After L929 and C6 cells were seeded into 96-well plates, respectively, 100 μL YHM DMEM solutions with different concentrations were added. After 24 h, the cell viability was calculated by CCK-8 assay.

**In vitro SDT**

After C6 cells were seeded into 96-well plate for 24h, the cells were treated with different ways: control, US, YH+US and YHM+US groups. After treated with US (0.7 W cm^-2^, 3 MHz, 50% duty cycle, 4 min, 1.0 cm thickness pork) for 5 h, the standard CCK-8 assay was used to evaluate the cell viability.

**In vivo SDT**

The tumor-bearing rats were randomly separated to 4 groups: (a) Control; (b) US; (c) YHM and (d) YHM+US. After YHM (5 mg mL^-1^, 600 μL) was injected for 6 hours, the groups (b) and (d) were treated with US (1.5 W cm^-2^, 3 MHz, 50% duty cycle, 8 min). T_2_-weighted MRI was used to assess the therapeutic effect of orthotopic gliomas. The body weight changes were continuously recorded after US treatment. The tumor volume was calculated by the formula (tumor volume = length × (width)^2^/2).

**Intracellular calcein-AM/PI staining**

C6 cells were co-cultured with YHM (200 ppm) for 24 h in a 24-well plate. The cells were treated with different ways: control, US, YH+US and YHM+US groups, and stained with calcein-AM/PI. Washed with PBS and collected images by fluorescent microscope.

**In vivo T_1_-weighted MRI**

YHM (5 mg mL^-1^, 600 μL) was injected into the rats (with gliomas) through tail vein, and T_1_-weight MRI images were collected using a 3.0 T MRI scanner.

**In vivo NIR-II imaging**

Vascular imaging: YHM (10 mg mL^-1^, 500 μL) was slowly injected into the C57BL/6J mouse through caudal vein with indwelling needle. With irradiation with 808 nm laser (5.0 W cm^-2^), the images were collected using NIR-Ⅱ imaging camera.

Orthotopic glioma imaging: YHM (10 mg mL^-1^, 500 μL) was injected into C57BL/6J mice, and then collected the images at 6 and 18 h post-injection.


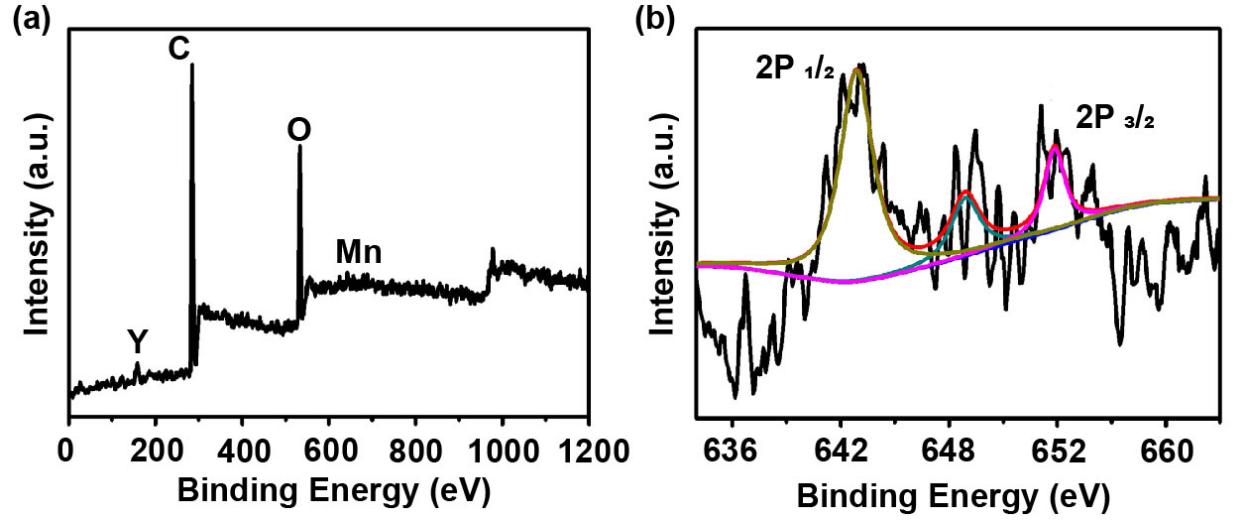


**Fig. S1 (a)** X-ray photoelectron spectroscopy (XPS) survey spectra of YHM; (**b)** Mn element XPS spectrum, together with their corresponding fitting curves.


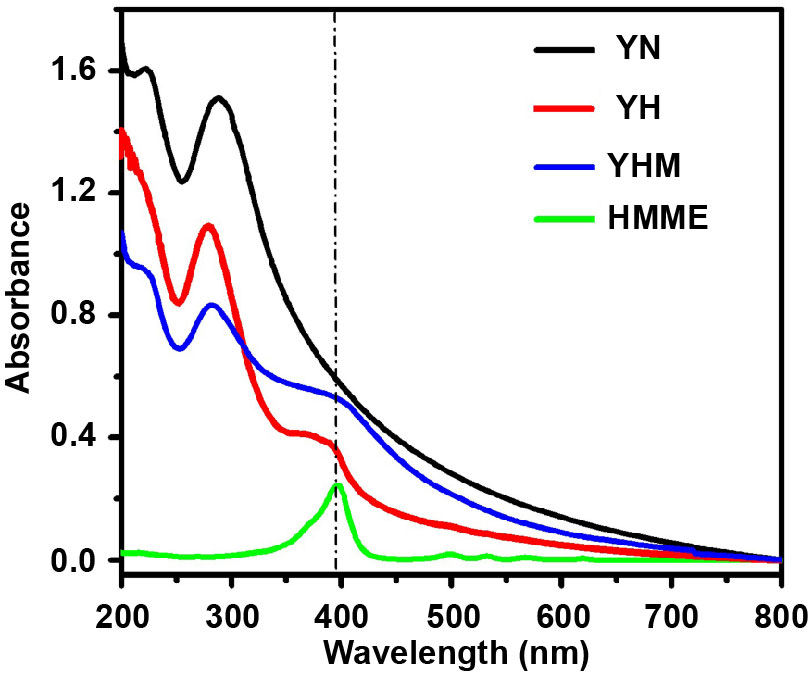


**Fig. S2** UV-vis absorbance spectra of YN, YH, YHM and HMME.


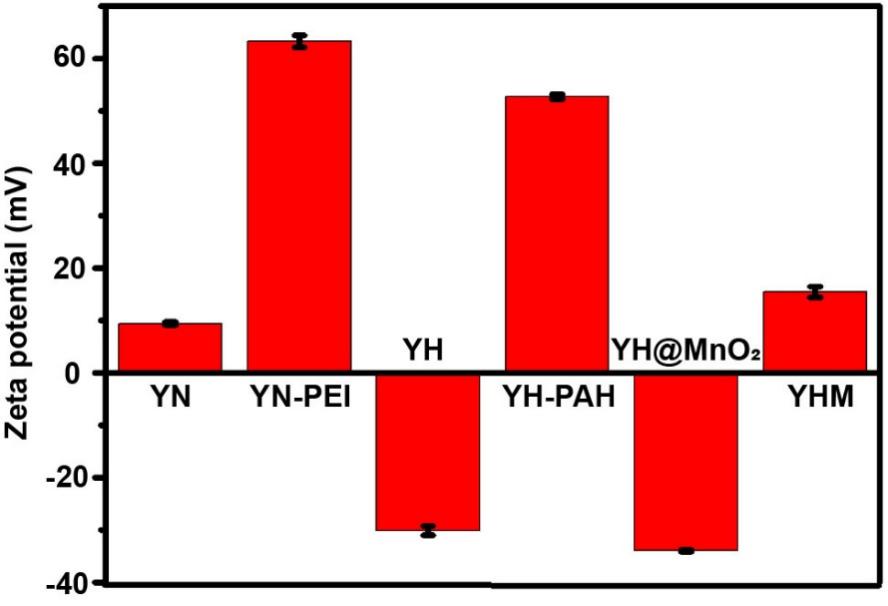


**Fig. S3** Zeta potential of YN, YN-PEI, YH, YH-PAH, YH@MnO_2_ and YHM.


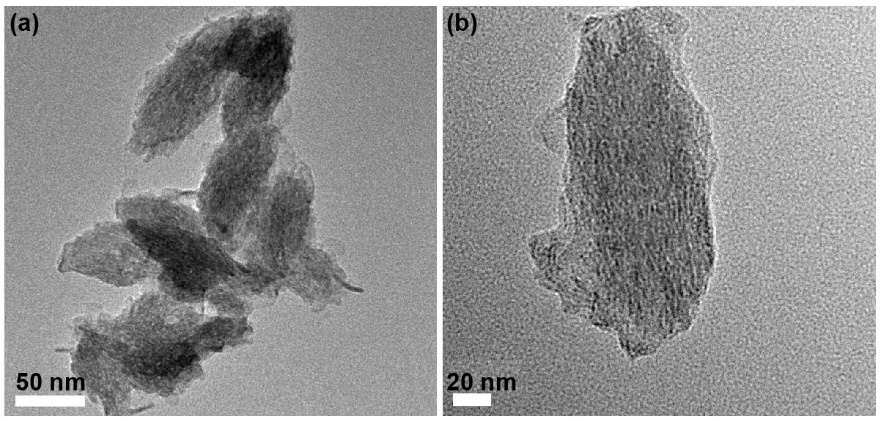


**Fig. S4** (**a**) TEM images of YHM mixed with phosphate buffered saline and (**b)** fetal bovine serum for 48 h.


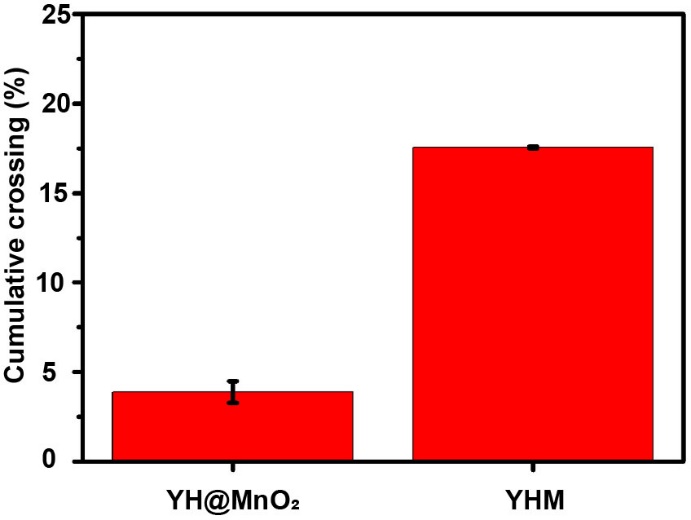


**Fig. S5** The BBB-crossing dynamics of YH@MnO_2_ and YHM for 24 h.


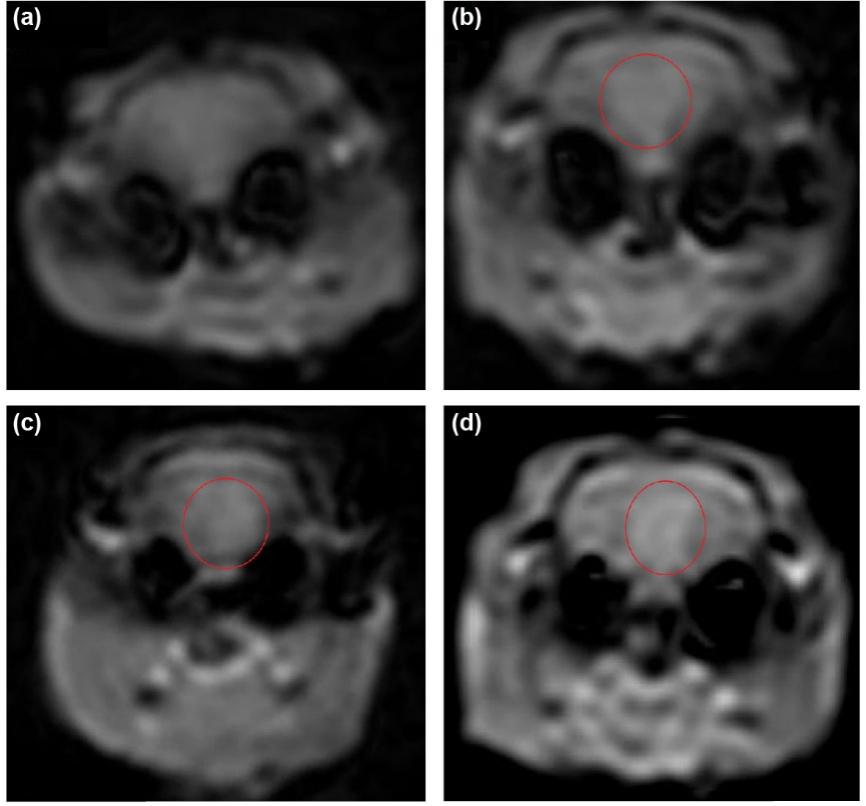


**Fig. S6** In vivo T_1_-weighted MRI imaging after YHM injected for 0 h (a), 3 h (b), 6 h (c) and 24 h (d).


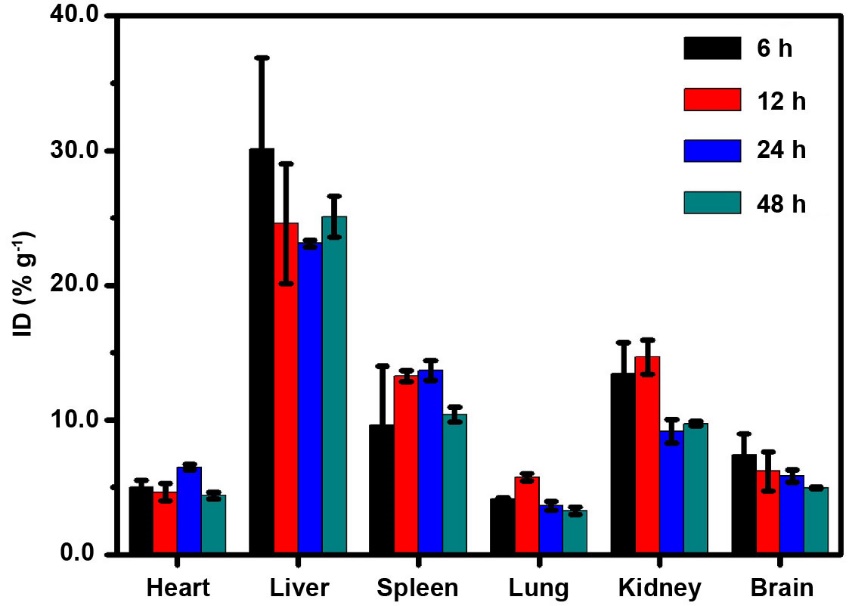


**Fig. S7** Biodistributions of Mn in tumor and main organs at different times (6, 12, 24 and 48 h).


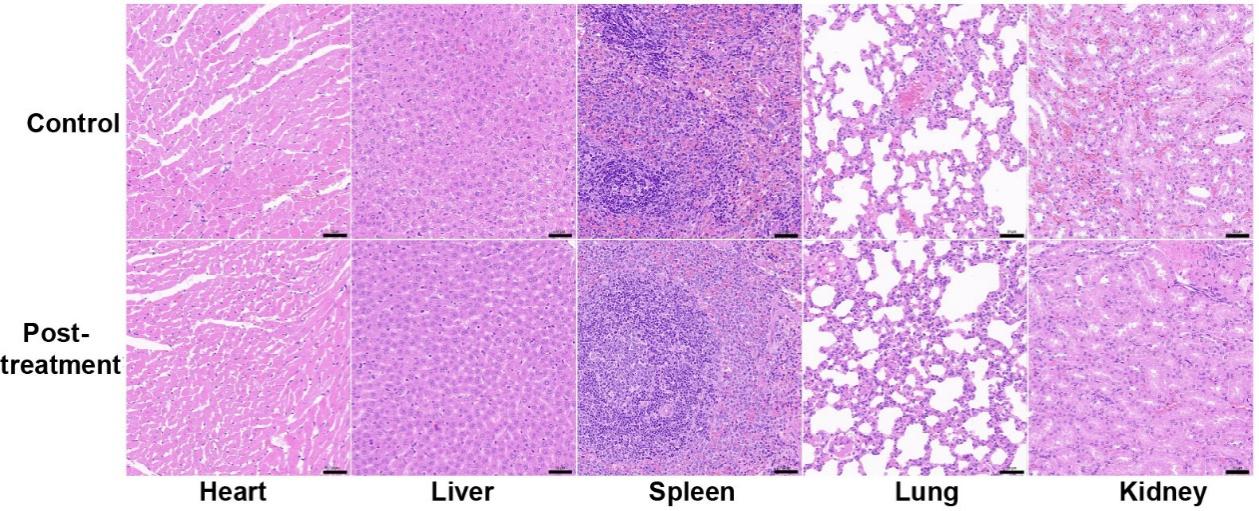


**Fig. S8** H&E stained images of control and posttreatment rats (scale bar: 50 µm).


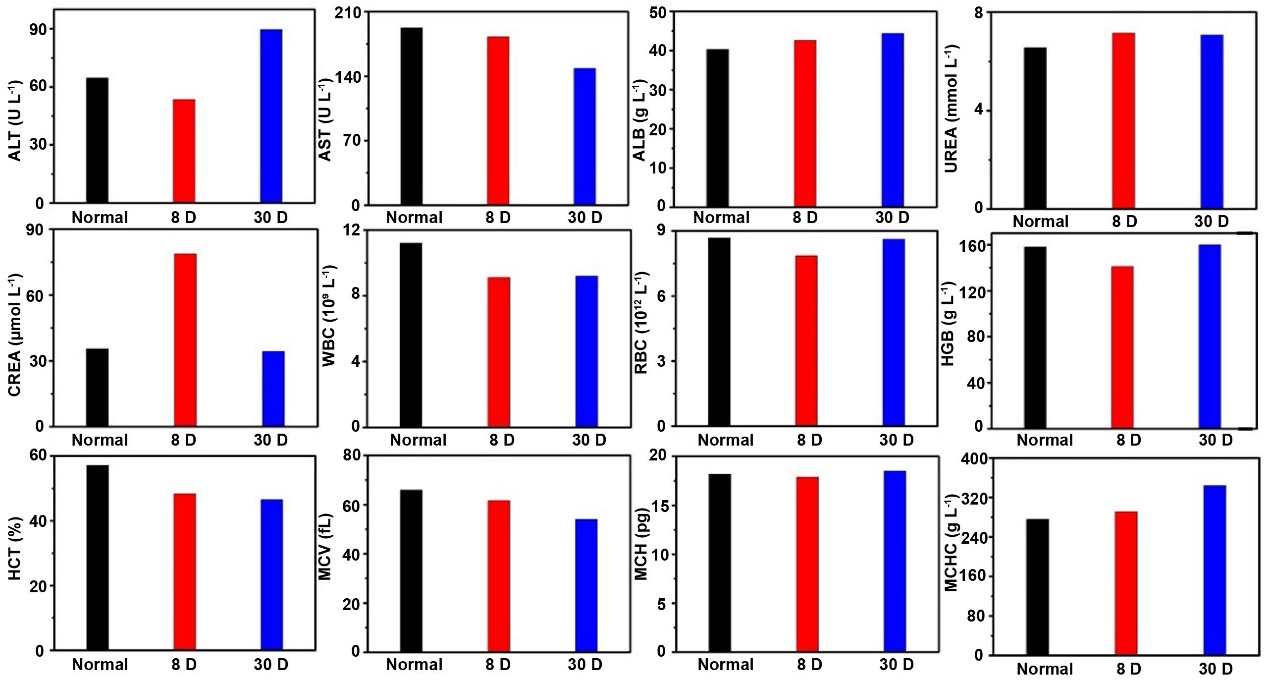


**Fig. S9** Blood biochemistry/complete blood panel analysis data of rats after intravenous injection of YHM collected at normal, 8 days and 30 days post-injecti­­­on (alanine aminotransferase (ALT), alkaline phosphatase (ALP), aspartate aminotransferase (AST), urea (UREA), creatinine (CREA), white blood cells (WBC), red blood cells (RBC), hemoglobin (HGB), hematocrit (HCT), mean corpuscular volume (MCV), mean corpuscular hemoglobin (MCH) and mean corpuscular hemoglobin concentration (MCHC).


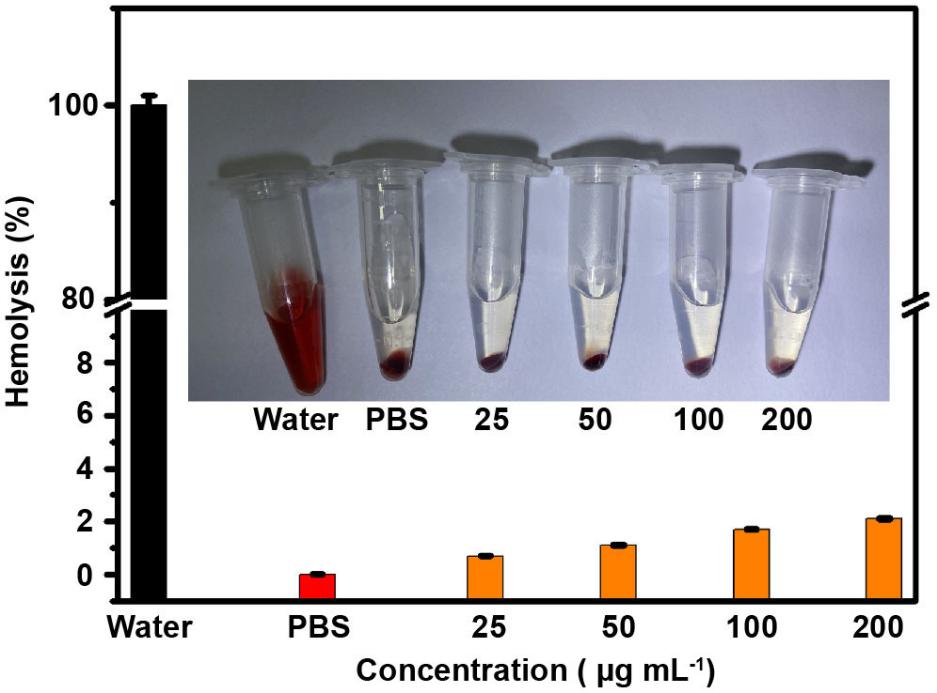


**Fig. S10** Hemolysisrate by incubating RBCs with DI water (positive control), PBS (negative control) or YHM NPs under various concentrations. (Inset: corresponding digital photos of centrifuge tube containing different samples).


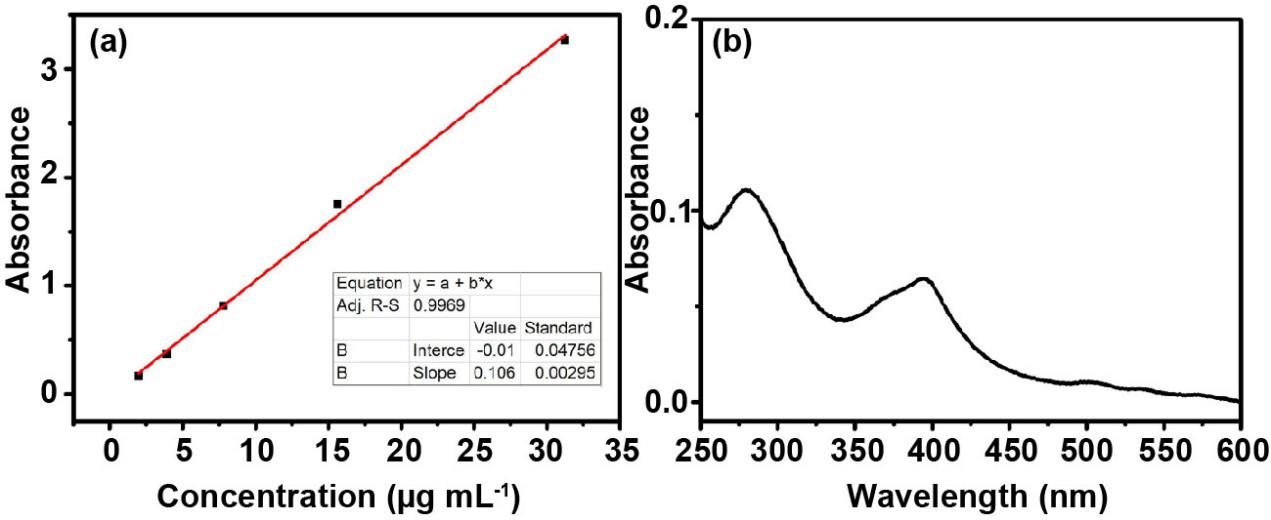


**Fig. S11** UV-vis absorbance spectra of (a) the standard curve of HMME and (b) supernatant of YVO_4_-HMME.
